# Clustering approaches for visual knowledge exploration in molecular interaction networks

**DOI:** 10.1186/s12859-018-2314-z

**Published:** 2018-08-29

**Authors:** Marek Ostaszewski, Emmanuel Kieffer, Grégoire Danoy, Reinhard Schneider, Pascal Bouvry

**Affiliations:** 1 Luxembourg Centre for Systems Biomedicine, University of Luxembourg, 7, Avenue des Hauts-Fourneaux, Esch-Belval, Luxembourg; 2 Interdisciplinary Centre for Security, Reliability and Trust, University of Luxembourg, 6, Avenue de la Fonte, Esch-Belval, Luxembourg; 3 Computer Science and Communications Research Unit, University of Luxembourg, 6, Avenue de la Fonte, Esch-Belval, Luxembourg

**Keywords:** Clustering, Bi-level optimization, Evolutionary algorithms, Molecular diagrams, Ontology, Knowledge discovery

## Abstract

**Background:**

Biomedical knowledge grows in complexity, and becomes encoded in network-based repositories, which include focused, expert-drawn diagrams, networks of evidence-based associations and established ontologies. Combining these structured information sources is an important computational challenge, as large graphs are difficult to analyze visually.

**Results:**

We investigate knowledge discovery in manually curated and annotated molecular interaction diagrams. To evaluate similarity of content we use: i) Euclidean distance in expert-drawn diagrams, ii) shortest path distance using the underlying network and iii) ontology-based distance. We employ clustering with these metrics used separately and in pairwise combinations. We propose a novel bi-level optimization approach together with an evolutionary algorithm for informative combination of distance metrics. We compare the enrichment of the obtained clusters between the solutions and with expert knowledge. We calculate the number of Gene and Disease Ontology terms discovered by different solutions as a measure of cluster quality.

Our results show that combining distance metrics can improve clustering accuracy, based on the comparison with expert-provided clusters. Also, the performance of specific combinations of distance functions depends on the clustering depth (number of clusters). By employing bi-level optimization approach we evaluated relative importance of distance functions and we found that indeed the order by which they are combined affects clustering performance.

Next, with the enrichment analysis of clustering results we found that both hierarchical and bi-level clustering schemes discovered more Gene and Disease Ontology terms than expert-provided clusters for the same knowledge repository. Moreover, bi-level clustering found more enriched terms than the best hierarchical clustering solution for three distinct distance metric combinations in three different instances of disease maps.

**Conclusions:**

In this work we examined the impact of different distance functions on clustering of a visual biomedical knowledge repository. We found that combining distance functions may be beneficial for clustering, and improve exploration of such repositories. We proposed bi-level optimization to evaluate the importance of order by which the distance functions are combined. Both combination and order of these functions affected clustering quality and knowledge recognition in the considered benchmarks. We propose that multiple dimensions can be utilized simultaneously for visual knowledge exploration.

**Electronic supplementary material:**

The online version of this article (10.1186/s12859-018-2314-z) contains supplementary material, which is available to authorized users.

## Background

Visual exploration of biomedical knowledge repositories is important for the users to handle their increasingly complex content. A significant amount of this content is encoded as graphs, representing known or inferred associations between bioentities of various types. Canonical pathway databases like KEGG [1], Reactome [2] or Wikipathways [3] provide small-scale, manually drawn diagrams of molecular mechanisms. Another type of repositories, like STRING [4], NDex [5] or SIGNOR [6], rely on large databases of associations, which are queried and visualized as graphs. These graphs are generated procedurally and rely on automated layout algorithms.

An important kind of knowledge repository combines the properties of pathway databases and association repositories. These are middle to large size molecular interaction diagrams, established in the context of systems biomedicine projects. Such diagrams are in fact knowledge maps, covering different areas from basic molecular biology [7–11] to various diseases [12–15]. Especially in the area of human diseases they offer contextualized insight into interactions between numerous convoluted factors like genetic profile, environmental influences or effects of medications.

In order to efficiently support health research, these knowledge maps have to be useful and interpretable for domain experts, like life scientists or medical doctors. This is a challenge, as the knowledge mapped into such diagrams is difficult to explore because of their size and complexity. This is well reflected by the fact that they need dedicated software to be used efficiently [16–18]. Recently proposed solutions suggest coloring of entire modules in such diagrams using experimental datasets [17, 19]. However, they rely on existing definitions of modules, introduced when the maps were drawn. New solutions for aggregating information are needed to enable the discovery of new knowledge from these established repositories.

In this paper we investigate the application of clustering to visual knowledge exploration in large molecular interaction maps. We propose to combine different distance functions to use prior information about curator’s expertise (Euclidean distance), network structure (graph distance) and higher-order associations between the elements (ontology distance). We demonstrate that clustering based on the combination of these functions yields more informative results, especially when the functions are combined using a novel bi-level optimization approach.

### Clustering in data exploration

With the emergence of online visual repositories like disease maps [14, 15] or metabolic maps [20], it becomes important to provide their users with high-order interpretation of the content. As these repositories are large and densely networked diagrams, their visual examination, especially for discovery and data interpretation purposes, is a challenging task. Clustering approaches are a plausible methodology to address the challenge of visual exploration and understanding of large, complex networks.

Clustering Analysis (CA) enables to discover relations between data points by grouping them following a defined similarity metric. It is a very important tool in biomedical data interpretation, as it allows to explore and mine high-dimensional datasets. As a number of CA methods are summarized and compared in a recent review [21], here we would like to focus on an important aspect of the problem, which is the application of similarity measures, in particular for graphs.

The literature is rich with clustering algorithms [22]. Since even for planar clustering the problem is NP-hard [23], i.e. it cannot be solved in polynomial time by a deterministic algorithm, the use of exact optimization solvers is clearly not suitable for large datasets. Thus, most clustering approaches are based on heuristics, including broadly recognized methods like k-means [24], k-medoids [25] and hierarchical clustering [26]. These and more sophisticated approaches rely on the notion of similarity, or a distance, between clustered objects, obtained using various distance metrics [27]. It is worth mentioning that although different similarity metrics in clustering were evaluated on the same datasets [28, 29], their combination for improved clustering accuracy was proposed only recently [30].

Distance functions can be used to define a grid in the data space, a paradigm used by grid clustering algorithms [31], detecting cluster shapes with a significant reduction of the computational complexity when considering large data sets. In turn, distribution models [32] estimate density for each cluster based on the distance between data points, allowing statistical inference of the clustering. An interesting approach is the Formal Concept Analysis [33], where a concept is an encoding extending the definition of distance or similarity. Generally, concepts allow to represent clusters with a set of satisfied properties, extending the criterion beyond distance. For instance, its application to disease similarity analysis [34] introduced a bipartite graph of disease-gene associations to define clusters of similar diseases.

As these heuristics may be trapped in local optima, alternatives based on evolutionary computing emerged recently. Genetic algorithms have shown their abilities to overcome the drawbacks encountered in basic clustering algorithms [35].

### Graph clustering in biomedicine

In biomedical research, disease mechanisms are often represented as networks of interactions on different scales - from molecular to physiological. These networks are in fact graphs, which can reach substantial size and complexity, as our knowledge on disease mechanisms expands. In order to make accurate interpretations using this interconnected body of knowledge, new approaches are needed to visualize meaningful areas and interactions in large biomedical networks.

Visual exploration of complex graphs requires certain aggregation of information about their content and structure, providing the user with an overview of dense areas of the graph, and their relationships. This task can be facilitated by means of graph clustering. Graph clustering groups vertices or edges into clusters that are homogeneous in agreement with a certain predefined distance function. An example is the application of local neighborhood measures to identify densely connected clusters in protein-protein interaction networks [36, 37]. Another approach is to construct clusters based directly on the global connectivity of the graph to identify strongly connected subgraphs [38, 39]. In these methods however, the visualization component of graph exploration is outside of the scope of analysis. Moreover, focusing on graph structure alone does not benefit from additional information on edges and vertices, available via various bioinformatics annotations. For instance, *eXamine* [40] uses annotations to improve the grouping of network elements for their better visualization, while MONGKIE [41] bases on clustering graph-associated ’omics’ data to improve the visual layout. Another interesting method, *Network2Canvas*, proposes a novel lattice-based approach to visualize network clusters enriched with gene-set or drug-set information. Importantly, the approaches discussed above focus either on large networks without a visual layout (protein-protein interaction networks) or on small-scale molecular diagrams. However, to the best of our knowledge, the challenge of clustering of large, manually curated molecular interaction diagrams [14] remains to be addressed.

In this work, we focus on graph clustering of large repositories of molecular interaction networks. As these not only carry the information about their graph structure, but also information about manual layout and annotation of the elements, we decided to explore the simultaneous use of multiple distance functions to create the clusters.

## Method

In this work we propose to combine different distance functions to improve the clustering results of large molecular interaction maps. We approach the problem by applying three distinct distance functions to the Parkinson’s and Alzheimer’s disease maps as our use cases. We then introduce and implement a bi-level clustering approach to obtain clustering from pairwise combinations of these metrics. We compare our algorithm against hierarchical clustering applied for the same set of distance functions. We evaluate the solutions by comparing against expert-provided groupings of the maps’ contents, and by enrichment analysis of the obtained clusters.

### Distance functions

Different distance functions can be applied to manually curate molecular interaction networks, reflecting distinct aspects of their contents. When clustering the contents of selected disease maps (see “Benchmark repositories” section), we considered the three following distances: Euclidean, network distance and ontology-based.

#### Euclidean distance

We calculated the Euclidean distance between elements of the maps by obtaining absolute values of (*x*,*y*) coordinates of elements of type *gene*, *mRNA* and *protein*. The rationale behind this distance function is that the distance between manually drawn elements reflects expert’s knowledge about their similarity.

#### Network distance

We calculated the network distance between elements of the maps by constructing a graph from the interactions of the elements of type *gene*, *mRNA* and *protein*. PD map and AlzPathway are encoded in SBGN [42], which is essentially a hypergraph - interactions with elements are allowed. We transformed such a hypergraph into a graph by replacing each multi-element interaction by a clique of pairwise interactions between all elements in this interaction. The network distance over the resulting graph is the set of pairwise shortest paths between all elements in the graph. For unconnected elements we set the distance to 2∗*max*(*shortest* *path*).

#### Ontology-based distance

We used the GOSemSim [43] method to calculate pairwise similarity between the elements of the maps within the Gene Ontology (GO). The distance (*d*) was calculated as *d*=1/(1+*similarity*). Three versions of the distance matrix were calculated, for Biological Process (GO BP), Cellular Compartment (GO CC) and Molecular Function (GO MF) were calculated.

### Bi-level clustering model

In this work, we consider medoid-based clustering, where medoids act as cluster representatives and clusters are built around them. Clustering based on *k* medoids has two types of decision variables:

$${\begin{aligned} x_{jj} = &\quad \left\{ \begin{array}{ll} 1 & \text{if } \text{element j becomes a cluster representative, i.e. a medoid} \\ 0 & \text{else.} \end{array}\right. \\ x_{ij} = &\quad \left\{ \begin{array}{ll} 1 & \text{if } \text{element i is assigned to cluster represented by medoid j} \\ 0 & \text{else.} \end{array}\right. \end{aligned}} $$

The objective function F represents the total distance from data to their respective medoids: $\sum \limits _{i}\sum \limits _{j} d_{ij} x_{ij}$. The k-median problem was proven to be an NP-hard problem [44].

Clustering is sensitive to different distance metrics and combining them may be beneficial. Thus, we propose a bi-level clustering model to leverage the use of different distance metrics. The proposed model enables the choice of medoids with a specific distance metric that can be different from the one used to assign data to clusters. Such an approach permits to prioritize these metrics.

Bi-level optimization problems have two decision steps, decided one after another. The leader problem is referred to as the “upper-level problem” while the follower problem is the “lower-level problem”. The order between the levels is important and its change provides a different optimal solution. This nested structure implies that a bi-level feasible solution necessitates a lower-level optimal solution and the lower-level problem is a part of the constraints of the upper-level problem.

We use bi-level optimization for the clustering problem by applying Bender’s decomposition to obtain two nested sub-problems that embed the same objective function. Then, we can define a Stackelberg game [45] between pairs of distance functions to explore their combined impact on the clustering performance. Model 1 describes the bi-level optimization model used for clustering.


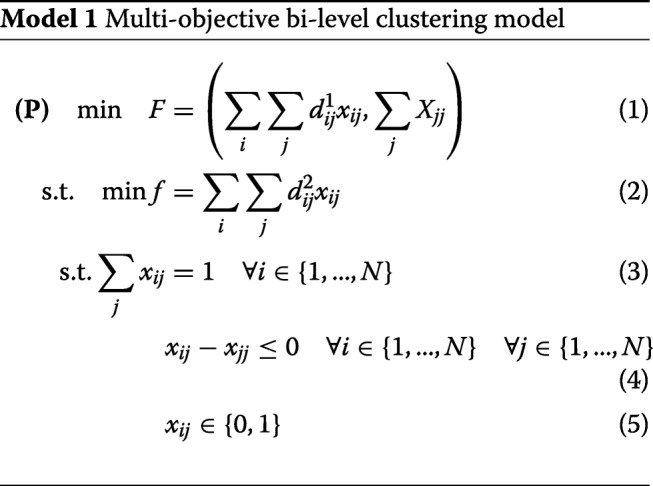


The term $\sum \limits _{i}\sum \limits _{j} d_{ij}^{1} x_{ij}$ represents the intra-class inertia due to the first distance function and the constraint $\sum \limits _{j} x_{jj} =k $ sets the number of clusters. The objective $\sum \limits _{i}\sum \limits _{j} d_{ij}^{2} x_{ij}$ is the intra-class inertia according to the second distance function. From constraint 3, only one data point should be only assigned to a single cluster while constraint 4 ensures that *j* becomes a cluster representative or medoid if any data point is assigned to it.

Regarding bi-level optimization, the variables *x*_*jj*_ are considered as upper-level decision variables while all variables *x*_*ij*_ such that *i*≠*j* are lower-level decision variables. Model 1 is in fact a decomposition of the original clustering problem. This allows us to set the cluster representatives with a first distance metric. Then, since these representatives are known, the lower-level problem is turned into an asymmetric assignment problem. In addition, lower-level decision variables *x*_*ij*_ will be automatically set to 0 in the case that *j* has not been selected as cluster representative. Even though the problem complexity did not change, i.e. it is still NP-hard, the decomposition allows to discover the polynomial part that can be solved exactly and efficiently, i.e. the assignment step.

The two objectives aim to minimize both the intra-class inertia and the number of clusters respectively. These are negatively correlated since the minimal intra-class inertia corresponds to as many clusters as data points, while a single cluster generates a maximal intra-class inertia. Thus, optimizing Model 1 results in a set of clusterings, which are alternatives or non-dominating solutions.

### Evolutionary optimization

Having defined the bi-level optimization model, we use the evolutionary algorithm approach to tackle the NP-hard clustering problem. A multi-objective evolutionary algorithm (MOEA) determines the best medoids at the upper-level with regards to the bi-objective vector $\min F = \left (\sum \limits _{i}\sum \limits _{j} d_{ij}^{1} x_{ij},\sum \limits _{j} x_{jj}\right)$ while an exact optimization algorithm is selected to optimize the lower-level problem $ \min \left \{ f\,=\,\sum \limits _{i}\sum \limits _{j} d_{ij}^{2} x_{ij}: \sum \limits _{j} x_{ij} \,=\,1 \!\quad \! \forall i \in \{1,...,N\}, x_{ij} \,-\, x_{jj} \leq 0 \forall i \in \{1,...,N \} \quad \forall j \in \{1,...,N \}{\vphantom {\left \{ f\,=\,\sum \limits _{i}\sum \limits _{j} d_{ij}^{2} x_{ij}: \sum \limits _{j} x_{ij} \,=\,1 \!\quad \! \forall i \in \{1,...,N\}, x_{ij} \,-\, x_{jj} \leq 0\right.}}\right \}$ where *x*_*ij*_,*x*_*jj*_∈{0,1}.

In Model 1, the medoids are represented by *x*_*jj*_, and once they are set, the lower-level problem becomes a classical assignment problem that can be solved optimally with a linear optimization algorithm (e.g., simplex, interior-point methods). This is due to the total unimodularity property of the constraint coefficient matrix when all *x*_*jj*_, i.e. upper-level decision variables are set.

This approach allows to create a bijection between a clustering and its total intra-class inertia. Indeed, we proceed in two phases as depicted by Algorithms 1 and 2. The MOEA initializes a population of clusterings. A clustering is a solution that is encoded using a binary vector indicating whether or not a data is considered as a medoid. Classical evolutionary operators are applied (see Table 1). However, in the proposed hybrid approach, the evaluation procedure differs from classical MOEAs. In order to evaluate a clustering, we create a linear assignment problem from the binary vector representing the selected medoids. All that remains is to solve exactly this problem in order to find out the best assignment of data to clusters.

**Table 1:** Experimental parameters

Parameters	
*Iterations*	30000
*Independent runs*	30
*Selection*	Binary tournament
*Crossover operator*	Single-point
*Crossover probability*	0.8
*Mutation operator*	Bit-flip
*Mutation probability*	$\frac {1}{Number~of~data}$
*Population size*	100


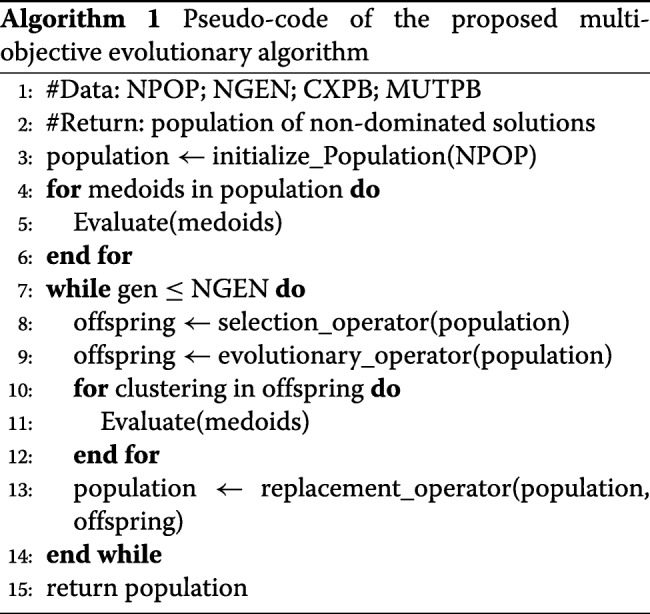



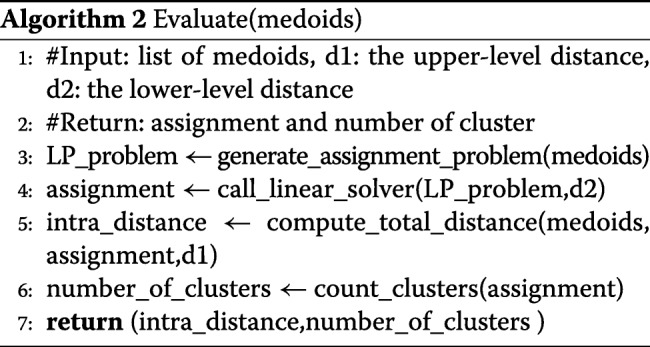


**Fig. 1:**
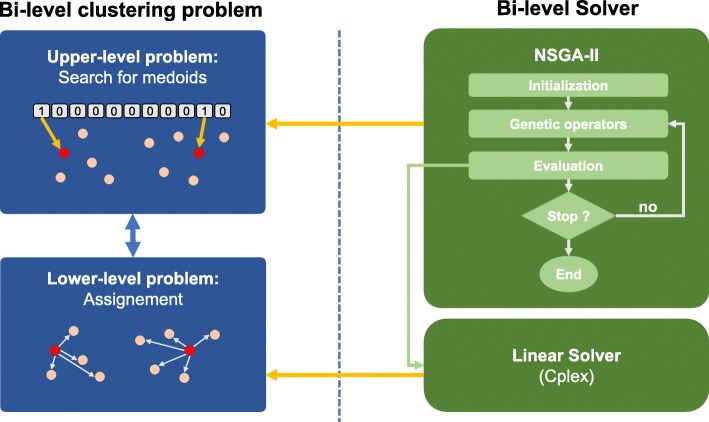
Bi-level optimization with GA. A scheme of our bi-level optimization approach. Clustering solutions are explored by GA based on the first optimization criterion, and evaluated with an exact solver for the second criterion

To solve the multi-objective problem we use the Non-dominated Sorting Genetic Algorithm (NSGA-II) [46]. As a linear exact solver we used the IBM ILOG CPLEX Optimizer’s mathematical programming technology [47], which is currently one of the most efficient solvers [48]. The general workflow of the hybrid algorithm is depicted in Fig. 1. Each generation of the algorithm involves standard evolutionary operators (see Algorithm 1), i.e. selection, crossover and mutation. The evolutionary algorithm iterated for 30000 generations in 30 independent runs in order to obtain good statistical confidence. Binary tournament was chosen as a selection method. We set the probability of a single-point crossover to 0.8, and the probability of a bit-flip mutation to $\frac {1.0}{Number~of~data}$. Concerning the CPLEX solver, no specific parameters have been selected. The stopping condition is the optimality of the solution. This is not an issue since the resulting assignment problem can be solved in polynomial time.

Each of the 30 independent runs returns a set of non-dominated solutions called Pareto front. Once the 30 runs have been performed, all fronts are merged together and the F-measure is computed for each solution. Since we are only interested in solutions with different clustering sizes and the merge operation can introduce duplicates, we filtered the solutions according the best F-measure.

Experiments have been conducted on the High Performance Computing platform of the University of Luxembourg [49]. The genetic algorithm has been implemented in Python with the DEAP library [50].

### Evaluation of clustering results

#### Benchmark repositories

We used two separate disease map repositories as evaluation datasets: the Parkinson’s disease map (PD map, pdmap.uni.lu) and the AlzPathway map (AlzPathway, alzpathway.org).

The PD map is a manually-curated repository about Parkinson’s disease, where all interactions are supported by evidence, either from literature or bioinformatic databases [14]. Similarly, the AlzPathway [12] is a map drawn manually on the basis of an extensive literature review about Alzheimer’s disease. Both diagrams are molecular interaction networks created in CellDesigner [51]. CellDesigner is an editor for diagrams describing molecular and cellular mechanisms for systems biology. It allows standardization and annotation of the content, which facilitates its analysis and reuse. Both PD map and AlzPathway were drawn by experienced researchers, based on extensive literature review on the known mechanisms of Parkinson’s and Alzheimer’s disease, respectively. The format of the diagrams, based on SBGN [42], allows to obtain the exact coordinates of the elements, their network structure and the annotations.

As both diagrams are human-drawn, the use of Euclidean distance is reasonable, as the clusters will reflect the curators’ knowledge. In turn, network and ontology-based distances will represent relationships difficult to comprehend by eye.

The PD map version from December’15 contains 2006 reactions connecting 4866 elements. Of these we selected 3056 elements of type *gene*, *mRNA* and *protein*. The AlzPathway (published version) contains 1015 reactions connecting 2203 elements, 1404 of which of type *gene*, *mRNA* and *protein* (see also “Method” section).

For these elements we extracted graphic coordinates for Euclidean distance and graph structure for network distance. For ontology-based distance, Entrez identifiers (www.ncbi.nlm.nih.gov/gene) are needed. For the PD map, HGNC symbols (www.genenames.org) were used to obtain Entrez ids. For the AlzPathway, Entrez ids were obtained from the Uniprot identifiers uniprot.org.

#### Benchmark for stability against content rearrangement

To test the robustness of our approaches in the situation when the content of a molecular interaction network changes, we prepared a reorganized version of AlzPathway (AlzPathway Reorg). The CellDesigner file for this new version is provided in the Additional file 1. The AlzPathway Reorg is rearranged in such a way that a number of nodes is duplicated, edge lengths are shortened and the content is grouped together locally. Overall, 225 new elements were added, 140 of which of type *gene*, *mRNA* and *protein*, and 16 reactions were removed as redundant. The resulting map in comparison to AlzPathway has an overall smaller Euclidean distance (0.372±0.183 vs 0.378±0.182) and bigger network distance (0.890±0.278 vs 0.601±0.420).

#### Expert-based evaluation

In order to evaluate the performance of the considered clustering approaches we applied expert-based, or external, evaluation. F-measure allows to assess how well the clustering is reflecting previously defined classes of data points [52]. We calculated the F-measure with *β*=5, also called F5 measure, using as target classes the annotation areas, e.g. “Mitophagy” or “Glycolysis”, available in the PD map and both versions of AlzPathway.

#### Discovery-based evaluation

The F-measure evaluates the performance of clustering in recreating previously defined groups, but is not capable of indicating how well a given set of clusters captures new knowledge. To evaluate the discovery potential of a given clustering solution we performed an enrichment analysis for GO [53] and Disease Ontology (DO) terms [54]. Similar evaluation was performed for annotation areas available in the PD map and both versions of AlzPathway, thus giving us a baseline for comparing expert-based organization of knowledge with different clustering approaches.

The enrichment analysis for both Gene and Disease Ontology was performed for each cluster separately, with all elements of the analyzed maps as background and adjusted *p*-value cutoff = 0.05, 0.01 and 0.001.

#### Benchmark clustering algorithm

All clustering results were compared against hierarchical clustering with grouping by Ward method [55], a popular clustering approach. To evaluate the combination of different distance functions, for each pair of distance functions we calculated the distance matrix *d*_pair_ as a product of the distance matrices normalized to the [−1,1] range. We used *d*_pair_ as the distance matrix for the hierarchical clustering algorithm.

## Results

### Combination of distance functions improves clustering quality

#### Hierarchical clustering

**Fig. 2:**
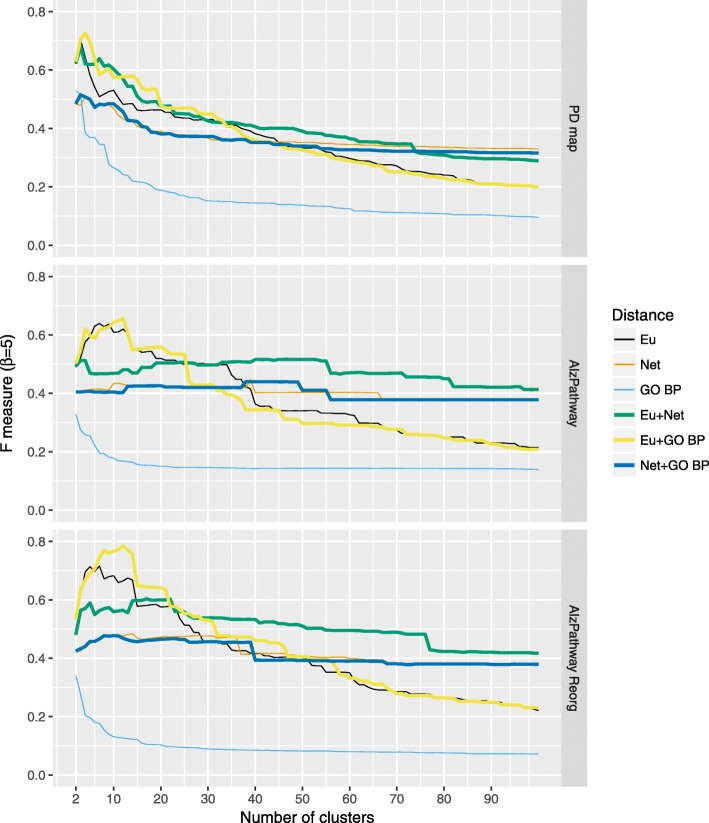
Hierarchical clustering (Ward) quality for different distance functions. The values of F-measure (*β*=5) for hierarchical clustering based on different distance functions and their pairwise combinations. Eu: Euclidean distance, Net: Network distance, GO BP: Gene Ontology-based (Biological Process) distance (for details see “Method” section)

**Fig. 3:**
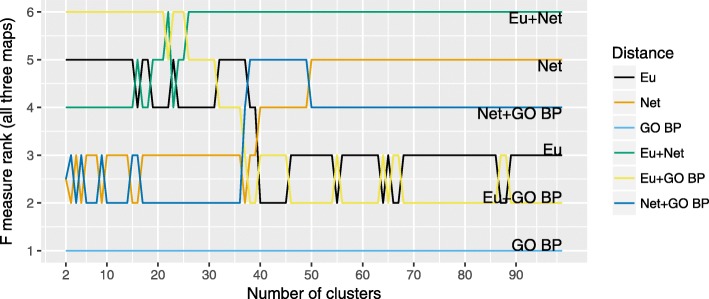
Ranking of different distance functions by summed F-measure for hierarchical clustering (Ward). Ranking of different distance functions and their pairwise combinations used with hierarchical clustering (Ward), by F-measure summed across three maps. Eu: Euclidean distance, Net: Network distance, GO BP: Gene Ontology-based (Biological Process) distance (for details see “Method” section)

We compared the quality of hierarchical clustering with Ward grouping (HCW) for three distance functions - Euclidean, network and Gene Ontology-based (Biological Process) - and their pairwise combinations on the contents of the PD map and two versions of AlzPathway (the original and the reorganized). For this purpose we applied expert-based evaluation to assess how well the clusters reflect the areas drawn in the maps to annotate groups of elements and interactions with a similar role. The results of our comparison are illustrated in Figs. 2 and 3, with Fig. 2 showing the particular F-measure scores for each map and distance metric. Figure 3 illustrates the ranking of particular distance metrics, constructed using F-measure summed for all three maps. Of three HCW with single distance functions, the Euclidean offers superior results over the other two for small cluster sets, while the network distance function is superior for larger sets. Pairwise combinations of distance metrics improve overall quality of clustering. Interestingly, Gene Ontology-based distance alone has the worst quality of clustering, but in combination with the Euclidean distance it improves the quality of smaller sets of clusters. Reorganization of the content, seen in comparison of two versions of AlzPatway, has a moderate effect on the quality of the clustering with a small improvement for cases with small number of clusters.

#### Bi-level clustering

**Fig. 4:**
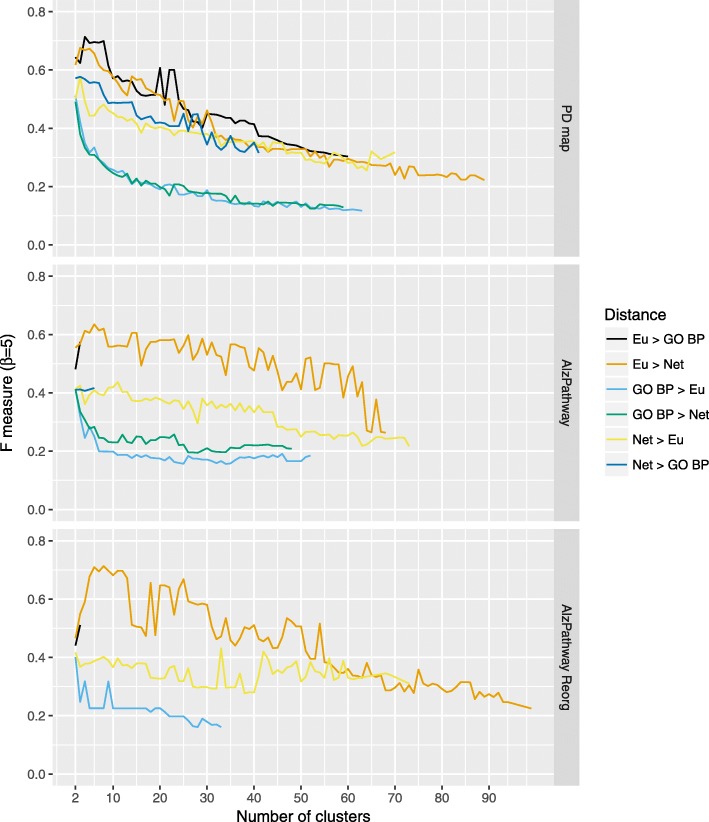
Bi-level clustering quality for different distance functions. The values of F-measure (*β*=5) for bi-level clustering based on pairwise combinations of distance functions, arranged as “leader” > “follower” distance functions, with Eu: Euclidean distance, Net: Network distance, GO BP: Gene Ontology-based (Biological Process) distance (for details see “Method” section)

**Fig. 5:**
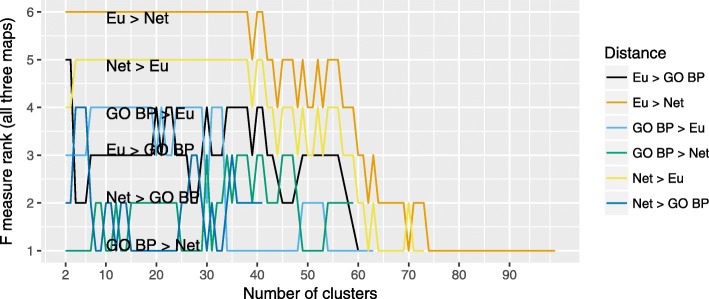
Ranking of different distance functions by summed F-measure for bi-level clustering. Ranking of different distance functions and their pairwise combinations used with bi-level clustering, by F-measure summed across three maps. Eu: Euclidean distance, Net: Network distance, GO BP: Gene Ontology-based (Biological Process) distance (for details see “Method” section)

Similarly, we calculated the F-measure for the results of bi-level clustering. The results are presented in Figs. 4 and 5. A comparison of the quality of different clusterings across the three maps shows grouping according to the “follower” distance function, with Gene Ontology-based metric being the worst-performing, and Euclidean being the best performing. As different combinations of distance functions yield varying number of clusterings, these pairings are the best observable in the PD map. For both instances of the AlzPathway there is either a small number, or no clusterings produced with GO BP metric as a follower. Reorganization of the content, seen in comparison of two versions of AlzPathway, has a bigger impact on the quality of the clustering than in the case of hierarchical clustering, where both combinations of GO BP and network distance no longer yield a viable clustering.

**Fig. 6:**
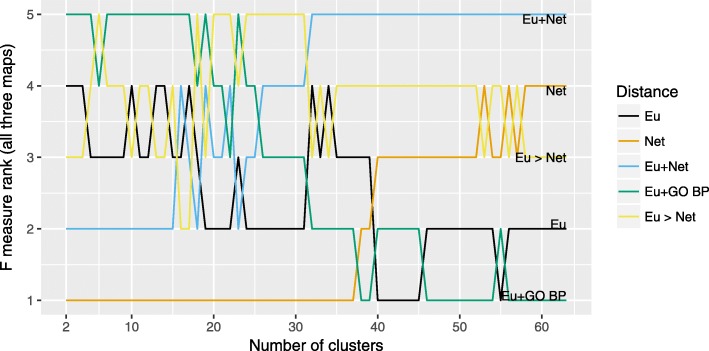
Ranking of Hierarchical (Ward) and Bi-level clustering approaches for selected distance functions. A combined ranking of the best performing distance functions (for hierarchical and bi-level clustering) by F-measure summed across three maps

A direct comparison of the best performing clustering schemes, as seen in Fig. 6, shows that HCW with the combined metrics offers the best F-measure values for the solutions with small and large number of clusters. The middle part of the clustering range (solutions between 20 and 30 clusters) is covered by the bi-level clustering (see Additional file 2).

### Bi-level clustering improves knowledge discovery

**Fig. 7:**
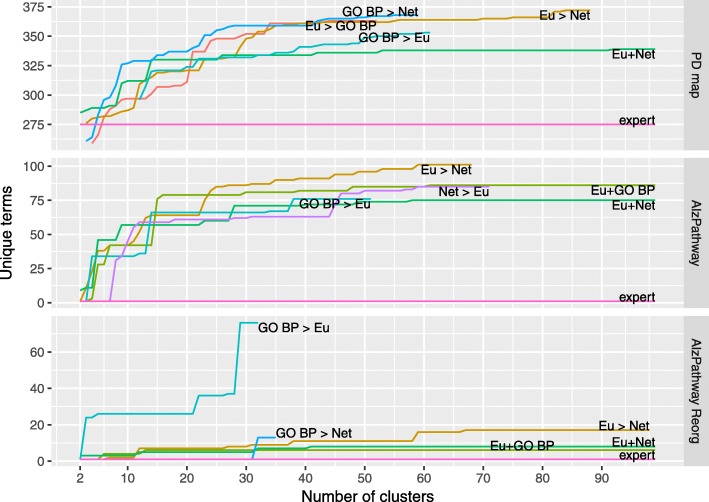
The comparison of hierarchical and bi-level clustering by discovered Disease Ontology. The number of Disease Ontology terms discovered by best performing bi-level and hierarchical clustering approaches. The curves represent the cumulative amount of unique terms enriched in all clusters in a given clustering. The adjusted *p*-value =0.001 was used as a cutoff threshold for the significance of an enriched term. For bi-level clustering, the distance functions are arranged “leader” > “follower”, with Euclidean: Euclidean distance, Net: Network distance, GO: Gene Ontology-based (Biological Process) distance (for details see “Method” section)

**Fig. 8:**
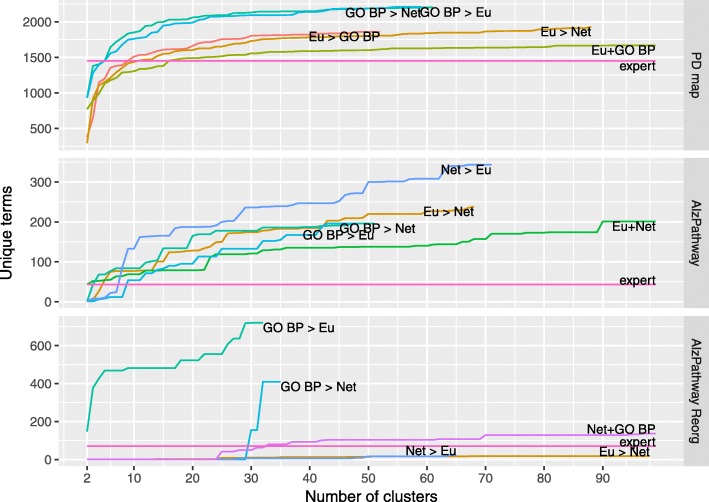
The comparison of hierarchical and bi-level clustering by discovered Gene Ontology terms. The number of Gene Ontology terms discovered by best performing bi-level and hierarchical clustering approaches. The curves represent the cumulative amount of unique terms enriched in all clusters in a given clustering. The adjusted *p*-value =0.001 was used as a cutoff threshold for the significance of an enriched term. For bi-level clustering, the distance functions are arranged “leader” > “follower”, with Euclidean: Euclidean distance, Net: Network distance, GO: Gene Ontology-based (Biological Process) distance (for details see “Method” section)

Next, we evaluated the impact of the bi-level clustering on discovery of new knowledge in comparison to HCW with combined distance functions. We performed an enrichment analysis for each set of clusters generated by each solution in the three maps. Each cluster was considered as a separate group of genes. We looked for enriched terms in Gene Ontology and Disease Ontology, with the cutoff threshold for adjusted *p*-value =0.001 (see “Method” section for more details). Figures 7 and 8 illustrate the results of our comparison for five best-performing approaches per map. With the same cutoff we calculated the enrichment of expert-provided annotation areas (“expert”) in the considered maps as a reference point to the performance of our clustering approaches.

The majority of proposed clustering approaches discover more unique terms than the expert-provided annotation for larger number of clusters. Notably, for the PD map both HCW and bi-level clustering approaches discovered more terms in the Disease Ontology than expert annotation for any number of clusters (Fig. 8). This also holds true for AlzPathway and AlzPathway Reorg, but given that only one DO term was discovered for expert annotation.

When comparing the performance of hierarchical and bi-level approaches, for larger number of clusters the bi-level clustering provides clusters enriched for more terms, both for Disease and Gene Ontology. Table 2 summarizes the highest scores for the selected clustering approaches. The table of complete results can be found in Additional file 3. For the PD map and AlzPathway maps, four out of five best distance metrics are bi-level solutions.

**Table 2:** Number of unique terms enriched in clusterings for different disease maps and ontologies (DO: Disease Ontology, GO: Gene Ontology)

	PD map		AlzPathway		AlzPathway Reorg	
Distance	DO	GO	DO	GO	DO	GO
Expert-based	36 / 275	36 / 1449	20 / 1	20 / 43	20 / 1	20 / 70
GO BP >Eu	**61 / 353**	**61 / 2203**	**51 / 76**	**51 / 196**	**32 / 76**	**32 / 720**
GO BP >Net	**59 / 368**	**59 / 2211**	48 / 72	**48 / 196**	**35 / 13**	**35 / 409**
Eu >GO BP	**52 / 363**	**52 / 1860**	3 / 5	3 / 19	3 / 1	3/1
Eu >Net	**88 / 372**	**88 / 1929**	**68 / 101**	**68 / 238**	**98 / 17**	**98 / 18**
Net >Eu	67 / 158	67 / 1463	**71 / 85**	**71 / 343**	65 / 1	**65 / 23**
Eu+Net	**93 / 339**	98 / 1641	**58 / 75**	**90 / 201**	**41 / 8**	2 / 1
Eu+GO BP	89 / 334	**97 / 1669**	**61 / 86**	97 / 179	**13 / 6**	90 / 14
Net+GO BP	81 / 289	86 / 1563	49 / 47	55 / 182	2 / 1	**97 / 136**

Each column contains a clusters/terms pair. Best values for each map are marked in bold

Interestingly, the bi-level clustering provides smaller number of clustering. This is due to the criterion in the evolutionary algorithm that stops further exploration of the search space if subsequent iterations offer no gain in the objective function. These results may suggest which distance functions offer better exploration of the search space and clustering properties.

When comparing AlzPathway and AlzPathway Reorg, one can notice that the restructuring of the map changed significantly the numbers of unique terms discovered, as well as ordering of the best performing combinations of metrics. However, bi-level clustering “GO BP > Eu” and “GO BP > Net” remained relatively stable with their amounts of discovered terms. Interestingly, the reorganization moderately reduced the amount of Disease Ontology terms, while significantly increasing the amount of Gene Ontology discovered terms.

We performed the enrichment analysis for higher adjusted *p*-value cutoffs : *p*−*adj*<0.05 and *p*−*adj*<0.1 (data not shown). We observed that the numbers of enriched terms for all clustering solutions as well as the expert-based one converge to the same levels.

### Examples of the discovered clusters

Here we discuss two examples of clustering results, also available in Additional file 4. Both examples come from bi-level clustering of the contents of the Parkinson’s disease map. Even though these distance pairs did not score high F-measures, their results reflect properly the content of the map and reveal new knowledge. To additionally validate the content of the clusters, we compared their content with the transcriptome of the brain area specific to Parkinson’s disease - the substantia nigra [56].

Example 1 is based on Euclidean-Network distances, scoring the highest for enrichment of the Disease Ontology terms. The cluster contains elements classified by experts as “Apoptosis” (Additional file 4: Box A), but also elements that by the original classification of the PD map belong to the “Glycolysis” area (Additional file 4: Box B). Interestingly, elements of Box B are known regulators of apoptosis in various contexts, including the neuronal environment with ENO1 [57] and SLC16A4 [58], and different types of cancer [59–61]. This can be considered as a novel regrouping of the content in the PD map, which would be difficult to discover optically, as the network distance between the elements of Box A and B cannot be immediately discerned by eye. When compared to the Parkinson’s disease transcriptome dataset, 19 out of 38 cluster elements were down-regulated, suggesting the importance of the contained mechanisms for the pathology of the disease.

Example 2 is based on Gene Ontology-Network distances, scoring the highest for enrichment of the Gene Ontology terms. When this cluster is displayed in the Parkinson’s disease map, it becomes evident that Euclidean distance was not used for its construction, as its elements are dispersed across the map. Nevertheless, the majority of the cluster contents are connected to the processes of response to oxidative stress and maintenance of mitochondrial homeostasis. There are, however, a number of elements that extend this picture. One of them is KCNN3, member of potassium calcium-activated channel family. Though originally curated in the map in the context of pathology of alpha-synuclein, its appearance in this cluster is supported by literature evidence [62]. Similarly, evidence supports inclusion of ATP13A2 in the mechanisms regulating oxidative stress [63]. On the other hand, the presence of GSK3A, another novel element, may be questionable. Even though its role in nerve regeneration was recently demonstrated [64], its association, together with PRKCD, may be due to the GO Biological Process annotation with cardiac myocyte function [65]. Still, when compared to the Parkinson’s disease transcriptome dataset, 94 out of 117 cluster elements were down-regulated, which gives confidence in its contents and corresponds well to the fact that reactive oxygen species play a major role in Parkinson’s disease [14].

### Gene ontology biological process is the most robust distance function in the evaluated scenarios

Three classification concepts are available in Gene Ontology: Biological Process, Cellular Compartment and Molecular Function. Thus, the ontology-based distance calculated according to these criteria may yield different results and, potentially, has different impact on the clustering results. Our metric of choice was Biological Process, as conceptually the closest to the nature of disease maps, describing processes of health and disease. To clarify the potential impact of the remaining concepts on the clustering quality, we compared clustering quality and enrichment of both hierarchical and bi-level approaches for all three. Figures Additional file 5 contain the results of this comparison.

F-measure values for hierarchical clustering are similar to each other, with GO BP having the highest impact on the clustering of the PD map, and GO CC on the AlzPathway Reorg. Nevertheless, this effect is rather moderate. Interestingly, the bi-level clustering results indicate that PD map and AlzPathway (original) could benefit from GO MF as the leader distance. Still, inclusion of these results would not alter the ranking of the distance metrics.

The number of enriched terms for Disease and Gene Ontology is also the highest for the BP-based ontology distance for PD map and AlzPahway Reorg. In case of the original AlzPathway, GO CC and MF as leader distances offer improvement in the discovered GO terms, but only for “GO MF > Eu” combination this improvement is noticeable. Overall, GO BP remains the most robust metric considered in our clustering analysis.

## Discussion

Large diagrams representing biomedical knowledge become an important part of workflows for interpretation of experimental data and generation of new hypotheses. Clustering approaches may provide a high-level overview of this complex content by grouping together similar elements. Different distance functions may be applied for this purpose. Here we investigated their impact on the clustering of the Parkinson’s disease (PD map) and Alzheimer’s disease (AlzPathway) maps.

First, we evaluated the impact of different distance functions on the clustering quality of the maps. We calculated the F-measure for HCW using expert-provided annotation areas in the PD map (see Fig. 2). Our results show an improvement when using combined distance functions, in particular Euclidean distance with Gene Ontology-based or network distances. Interesting is the contribution of the Gene Ontology-based distance. By itself this distance function has the lowest F-measure scores. When combined with the Euclidean distance it improves the F-measure beyond the performance of the Euclidean distance alone. This suggests that clustering based on combined distance functions may improve the quality of clustering results.

Next, in order to investigate the relationships between different distance functions we performed a bi-level clustering for the pairwise combinations of the considered distance metrics (see Fig. 3). The results are clearly grouped by the “follower” metric, with the Euclidean distance scoring the highest, and improving the performance of the HCW. Additionally, because of the stopping criterion in the evolutionary algorithm, the “leader” Gene Ontology-distance provides smaller sets of clusters. This is understandable, as the Gene Ontology-based distance describes the conceptual similarity between the contents of the map and has no reflection of the actual structure of the diagram. In turn, the expert-based annotations reflect visual areas of disease maps. Therefore, Gene Ontology-based distance will not perform well to define meaningful cluster medoids in the maps.

Finally, we evaluated the impact of combined distance functions on knowledge discovery in the maps. For each set of clusters from both HCW and bi-level clustering, we performed an enrichment analysis for Disease Ontology and Gene Ontology terms. Our results showed that the number of unique terms for both ontologies grows with growing size of cluster sets and surpasses the expert-provided annotation areas. Notably, if the number of expert-provided areas are taken as the cluster set size (36 in the PD map, 20 in AlzPathway and AlzPathawy Reorg), all but one selected clustering solutions provide more unique terms for the Disease Ontology. For enrichment in Gene Ontology terms in the reorganized AlzPathway, the methods are not as robust, but the “GO BP > Eu” bi-level clustering still offers a significant improvement over the expert-based annotation. These results, in combination with F-measure results, suggest that the results of these clustering approaches may offer an improvement to the existing annotation of the maps.

Bi-level clustering in direct comparison with HCW produces cluster sets with the overall lower score in F-measure, but higher number of enriched terms. In effect, both approaches may be a viable support to exploration of complex molecular interaction diagrams: bi-level in discovery of novel connections, hierarchical for better visual representation of clusters.

A comparison of different disease maps, including reorganizing content of AlzPathway, shows that local rearrangement of elements may have an impact on the number of enriched terms in the clusters. Interestingly, while the maximum number of Disease Ontology terms dropped moderately, the maximum number of Gene Ontology terms increased significantly. From this analysis, “GO BP > Eu” bi-level approach seems to be the most robust across both ontologies, however these results will have to be validated on more maps.

The study has certain caveats, which may affect the conclusions of the article. First, the F-measure evaluation depends on the expert annotation and a thorough analysis against a set of such annotations is needed to provide a better insight into the combination of distance metrics and their recall capabilities. Second, the results of the evolutionary algorithm are combined over a number of independent iterations and depend on a predefined set of parameters. Exploration of this parameter space is necessary to better evaluate the performance of the approach. Especially a detailed analysis of the impact of different parameters on ontology distance, e.g. required evidence or method for combining the similarity score, may bring further insight into improvement of the results of the algorithm. Finally, other disease maps may be analyzed in a similar way for a better understanding on how clustering may improve the usefulness of such repositories. Our focus was on Parkinson’s and Alzheimer’s disease, which may introduce bias to the analyzed results.

## Conclusions

In this paper we demonstrated the utility of combining different distance functions to meaningfully cluster the contents of a complex visual repository on human disease. We proposed a bi-level clustering approach as a solution for combining two distance functions and exploring their relationship. The cluster sets discovered by our approach reflect well the existing annotations of the PD map and are enriched for a higher number of unique terms in Disease and Gene Ontologies. Our solution offers an improvement to the process of exploration of complex biomedical repositories, e.g. disease maps. The experts can be aided by clustering results in annotation of high-level areas of such maps, increasing their clarity and helping in using their contents.

## Additional files

Additional file 1 Reorganized AlzPathway. This file contains a CellDesigner format of the reorganized AlzPathway, with a number of elements duplicated and rearranged to reduce their Euclidean distance while increasing their network distance. Original AlzPathway was published in [12], under the CC BY 3.0 license. See also alzpathway.org for details. (ZIP 261 KB)

Additional file 2 Complete F-measure results. This file contains a table with F-measure values for all tested clustering solutions. Additional file 5 contains a figure with comparison of maximum F-measure per clustering, per approach - hierarchical clustering with single distance function, hierarchical clustering with combined distance functions and bi-level clustering. (XLSX 197 KB)

Additional file 3 Complete enrichment results. This file contains a table with numbers of enriched terms for all tested clustering solutions. Additional file 5 contains a figure with comparison of all clustering solutions, per approach - hierarchical clustering with combined distance functions and bi-level clustering. (XLSX 215 KB)

Additional file 4 Bi-level clustering examples. This file contains a figure with selected examples of bi-level clustering. (PDF 551 KB)

Additional file 5 Comparison of GO distance functions. This file contains figures comparing F-measure and the number of enriched terms for different Gene Ontology distance functions. (PDF 125 KB)

## Abbreviations

AlzPathway: Alzheimer’s disease map
CA: Clustering analysis
DO: Disease ontology
GO: Gene ontology
GO BP: Biological process
GO CC: Cellular compartment
GO MF: Molecular function
HCW: Hierarchical clustering with ward grouping
MOEA: Multi-objective evolutionary algorithm
NSGA-II: Non-dominated sorting genetic algorithm
PD map: Parkinson’s disease map
