# Supplementary material for: Clustering approaches for visual knowledge exploration in molecular interaction networks
Source: BMC Bioinformatics. 2018 Aug 29;19:308. doi: 10.1186/s12859-018-2314-z (PMC6116538; doi:10.1186/s12859-018-2314-z)
Supplement: Supplementary file 4 — Bi-level clustering examples. This file contains a figure with selected examples of bi-level clustering. (PDF 551 KB) [file 12859_2018_2314_MOESM4_ESM.pdf]

Supplementary figure: Cluster example 1, Eu > Net bilevel clustering

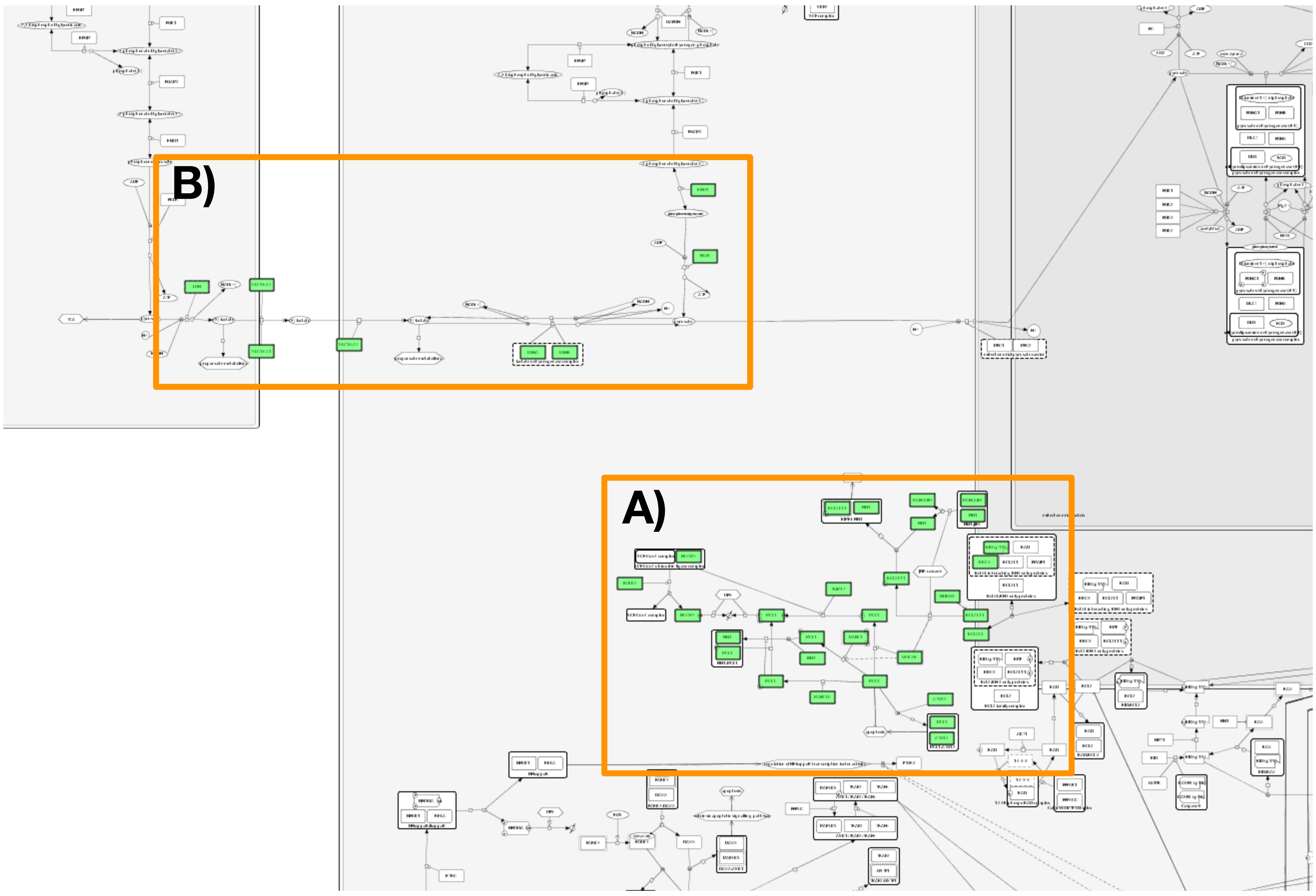

Elements belonging to the cluster (unique names, 38 total):

Box A) ATG12, BBC3, BCL2L1, BCL2L11, BID (p15), FBXW7, GSK3B, MAPK1, MAPK10, MAPK8IP3, MCL1, NDRG1, PARK2, PIN1, TRIM17

Box B) ENO1, LDH, LDHA, LDHB, PKLR, SLC16A1, SLC16A4, SLC16A7

Supplementary figure: Cluster example 2, GO BP > Net bilevel clustering

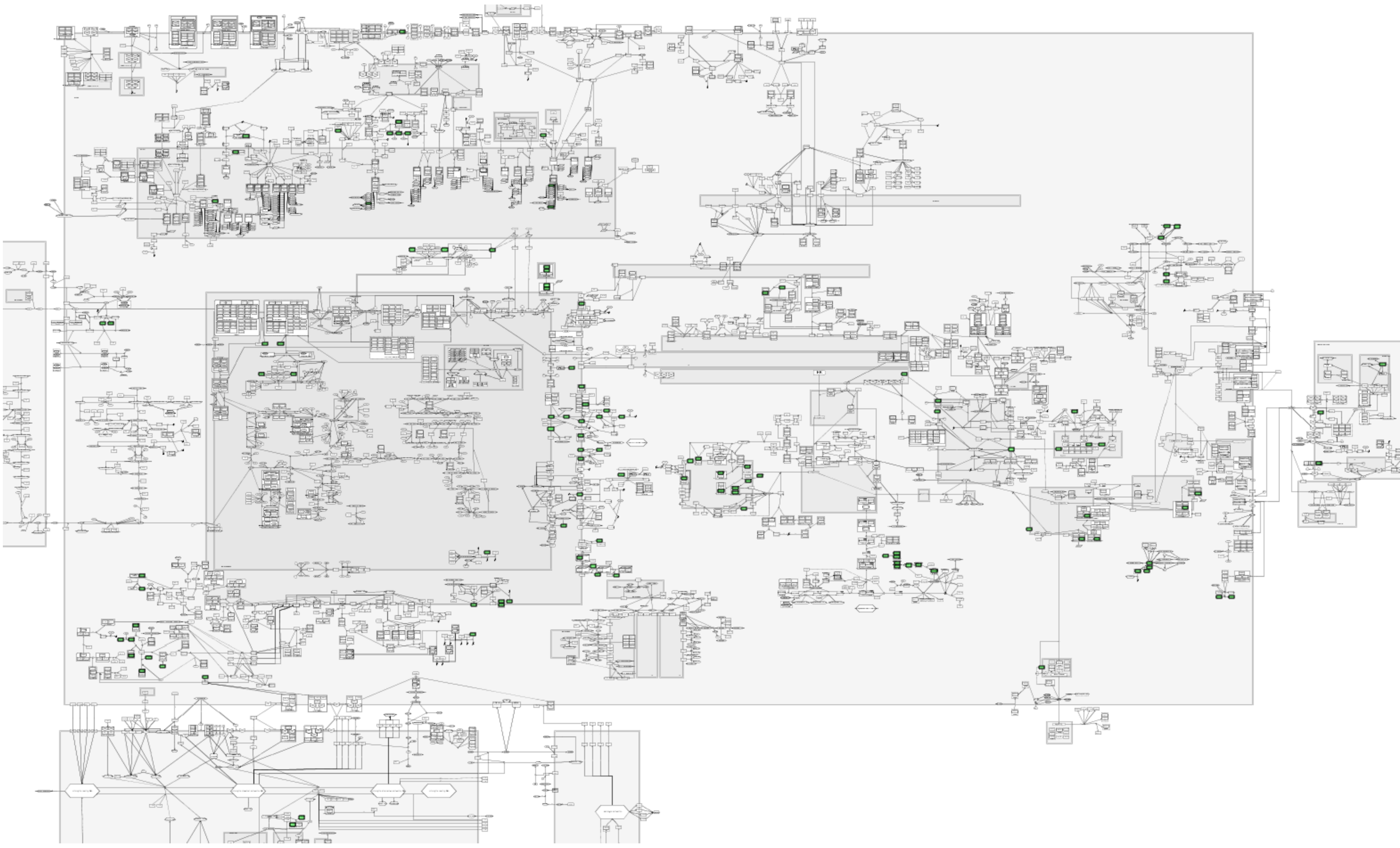

Elements belonging to the cluster (unique names, 117 total):

ATP13A2, BAG5, FBXW7, GBA, GSK3A, HAX1, KCNN3, MUL1, PARK7, PARL, PINK1, PRKCD, TOMM6, TXN, VPS35
