# Supplementary material for: Clustering approaches for visual knowledge exploration in molecular interaction networks
Source: BMC Bioinformatics. 2018 Aug 29;19:308. doi: 10.1186/s12859-018-2314-z (PMC6116538; doi:10.1186/s12859-018-2314-z)
Supplement: Supplementary file 5 — Comparison of GO distance functions. This file contains figures comparing F-measure and the number of enriched terms for different Gene Ontology distance functions. (PDF 125 KB) [file 12859_2018_2314_MOESM5_ESM.pdf]

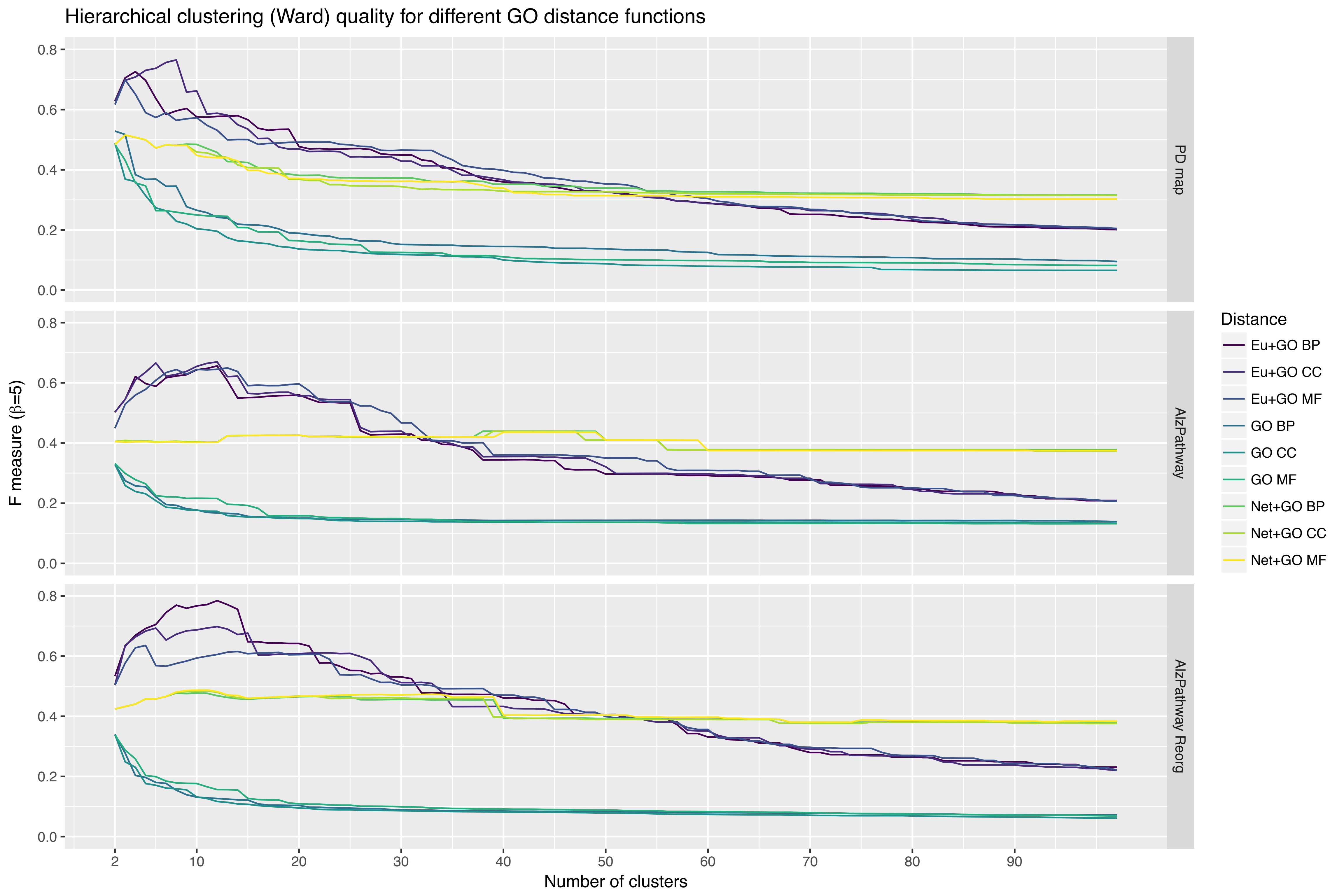

Hierarchical clustering (Ward) quality for different GO distance functions
PD map
AlzPathway
AlzPathway Reorg
F measure (β=5)
Number of clusters
0.0
0.2
0.4
0.6
0.8
2
10
20
30
40
50
60
70
80
90
Distance
Eu+GO BP
Eu+GO CC
Eu+GO MF
GO BP
GO CC
GO MF
Net+GO BP
Net+GO CC
Net+GO MF

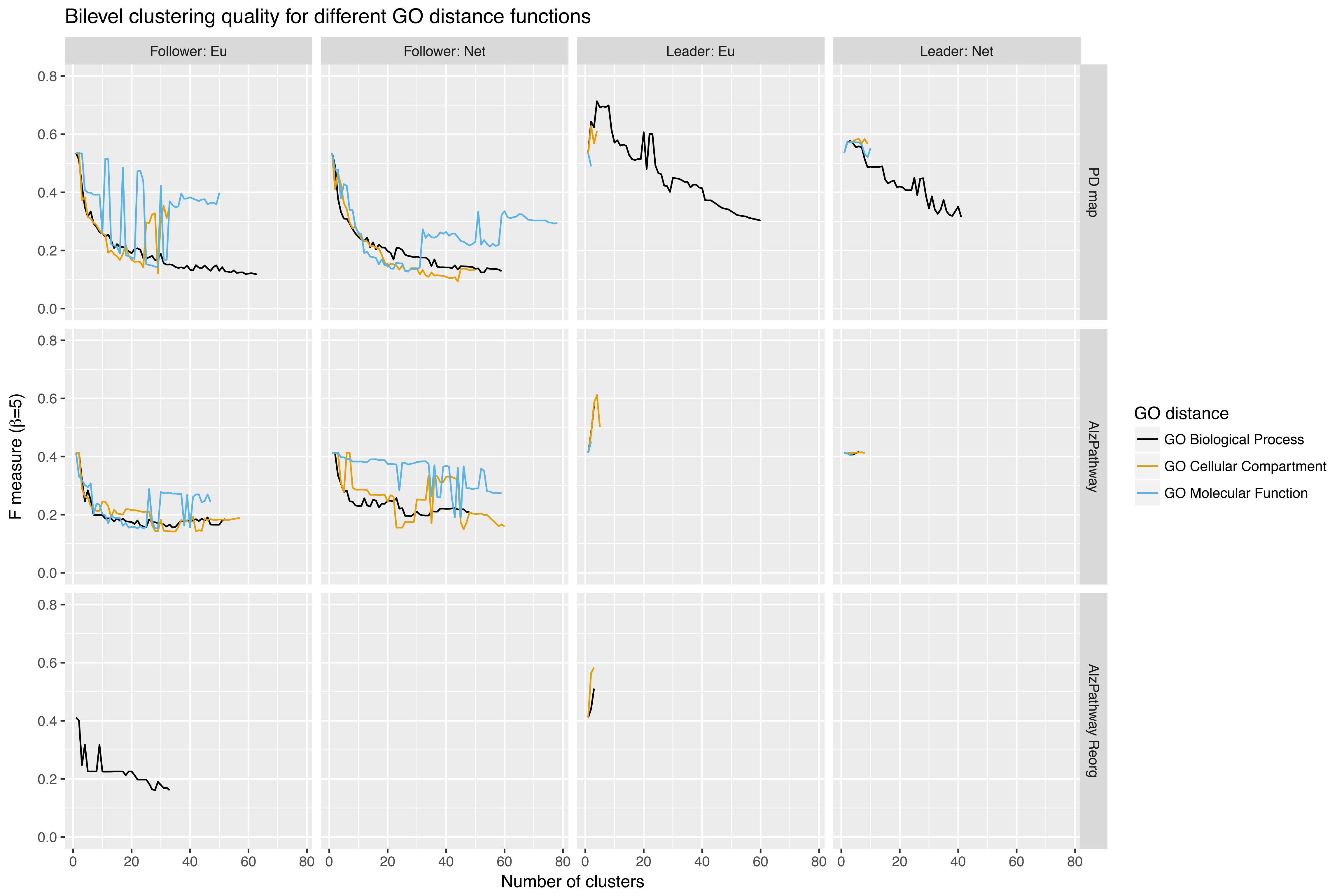

Bilevel clustering quality for different GO distance functions
Follower: Eu
Follower: Net
Leader: Eu
Leader: Net
PD map
AlzPathway
AlzPathway Reorg
F measure (β=5)
Number of clusters
0.0
0.2
0.4
0.6
0.8
0
20
40
60
80
GO distance
GO Biological Process
GO Cellular Compartment
GO Molecular Function

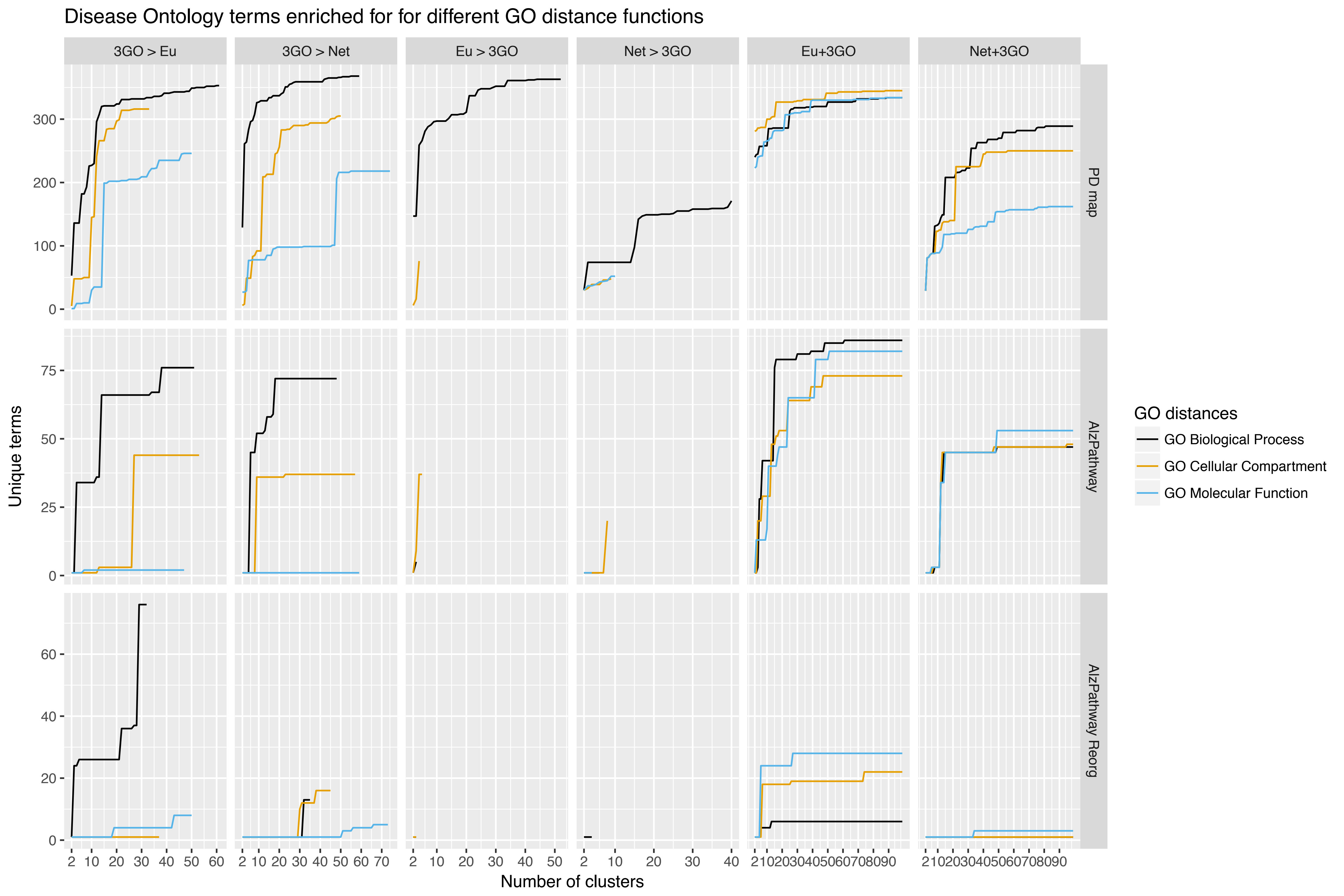

Disease Ontology terms enriched for for different GO distance functions
3GO > Eu
3GO > Net
Eu > 3GO
Net > 3GO
Eu+3GO
Net+3GO
PD map
AlzPathway
AlzPathway Reorg
Unique terms
Number of clusters
GO distances
GO Biological Process
GO Cellular Compartment
GO Molecular Function

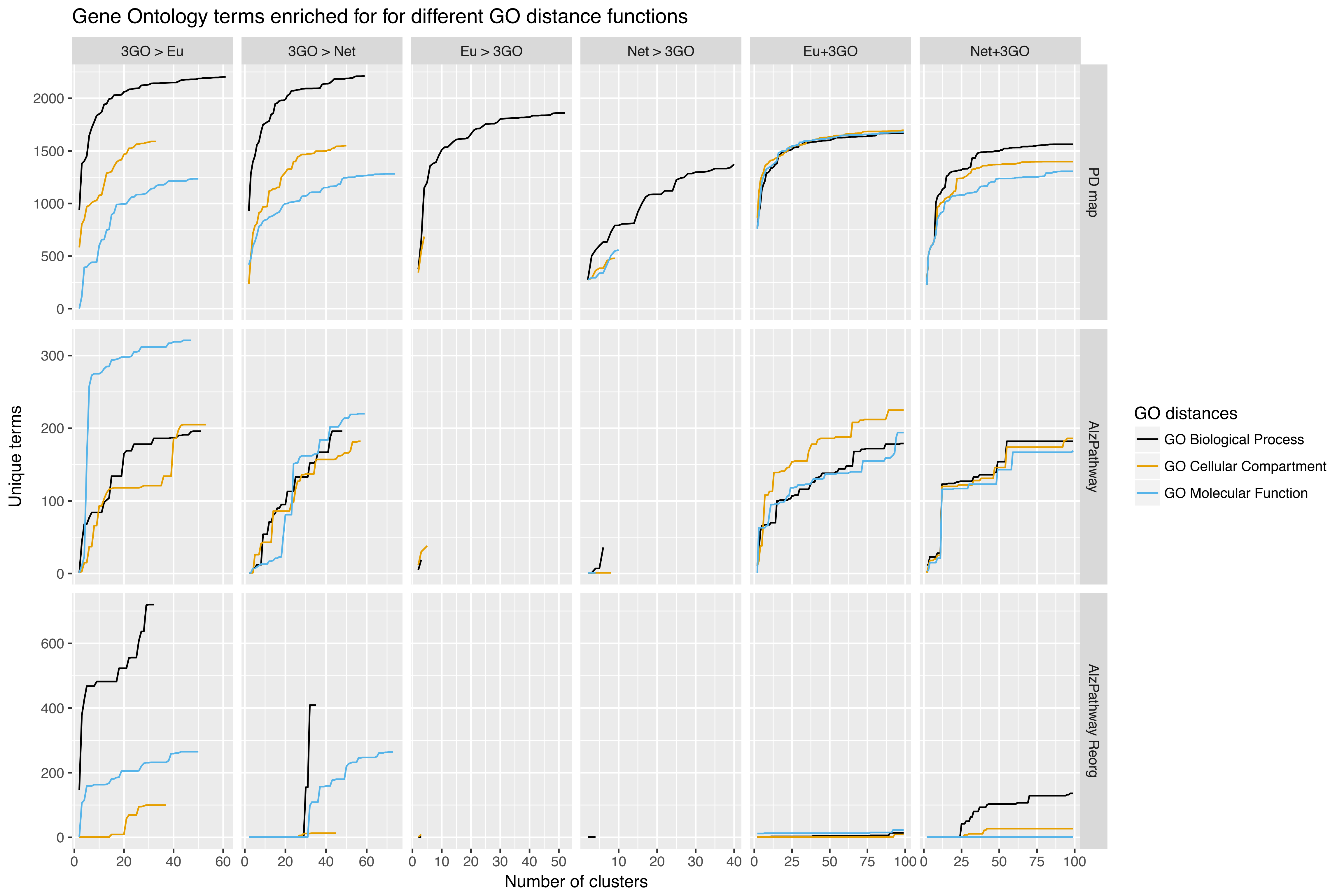

Gene Ontology terms enriched for for different GO distance functions
3GO > Eu
3GO > Net
Eu > 3GO
Net > 3GO
Eu+3GO
Net+3GO
PD map
AlzPathway
AlzPathway Reorg
Unique terms
Number of clusters
GO distances
GO Biological Process
GO Cellular Compartment
GO Molecular Function
